# Effect of Thermal Stress on Tissue Ultrastructure and Metabolite Profiles During Initiation of Radiata Pine Somatic Embryogenesis

**DOI:** 10.3389/fpls.2018.02004

**Published:** 2019-01-17

**Authors:** Ander Castander-Olarieta, Itziar A. Montalbán, Eliana De Medeiros Oliveira, Emilia Dell’Aversana, Luisa D’Amelia, Petronia Carillo, Neusa Steiner, Hugo Pacheco De Freitas Fraga, Miguel Pedro Guerra, Tomás Goicoa, María Dolores Ugarte, Catia Pereira, Paloma Moncaleán

**Affiliations:** ^1^Centro de Arkaute, Neiker-Tecnalia, Vitoria-Gasteiz, Spain; ^2^Central Laboratory of Electron Microscopy, Universidade Federal de Santa Catarina, Florianópolis, Brazil; ^3^Department of Environmental, Biological and Pharmaceutical Sciences and Technologies, Università degli Studi della Campania Luigi Vanvitelli, Naples, Italy; ^4^Department of Botany, Universidade Federal de Santa Catarina, Florianópolis, Brazil; ^5^Department of Botany, Universidade Federal do Parana, Curitiba, Brazil; ^6^Laboratório de Fisiología do Desenvolvimento e Genética Vegetal, Universidade Federal de Santa Catarina, Florianópolis, Brazil; ^7^Department of Statistics, Computer Science and Mathematics, Universidad Pública de Navarra, Pamplona, Spain; ^8^Department of Life Sciences, Universidade de Coimbra, Coimbra, Portugal

**Keywords:** amino acids, *Pinus radiata*, proteins, somatic embryo, sugars, temperature, transmission electron microscopy

## Abstract

Climate change will inevitably lead to environmental variations, thus plant drought tolerance will be a determinant factor in the success of plantations and natural forestry recovery. Some metabolites, such as soluble carbohydrates and amino acids, have been described as being the key to both embryogenesis efficiency and abiotic stress response, contributing to phenotypic plasticity and the adaptive capacity of plants. For this reason, our main objectives were to evaluate if the temperature during embryonal mass initiation in radiata pine was critical to the success of somatic embryogenesis, to alter the morphological and ultrastructural organization of embryonal masses at cellular level and to modify the carbohydrate, protein, or amino acid contents. The first SE initiation experiments were carried out at moderate and high temperatures for periods of different durations prior to transfer to the control temperature of 23°C. Cultures initiated at moderate temperatures (30°C, 4 weeks and 40°C, 4 days) showed significantly lower initiation and proliferation rates than those at the control temperature or pulse treatment at high temperatures (50°C, 5 min). No significant differences were observed either for the percentage of embryogenic cell lines that produced somatic embryos, or for the number of somatic embryos per gram of embryonal mass. Based on the results from the first experiments, initiation was carried out at 40°C 4 h; 50°C, 30 min; and a pulse treatment of 60°C, 5 min. No significant differences were found for the initiation or number of established lines or for the maturation of somatic embryos. However, large morphological differences were observed in the mature somatic embryos. At the same time, changes observed at cellular level suggested that strong heat shock treatments may trigger the programmed cell death of embryogenic cells, leading to an early loss of embryogenic potential, and the formation of supernumerary suspensor cells. Finally, among all the differences observed in the metabolic profile, it is worth highlighting the accumulation of tyrosine and isoleucine, both amino acids involved in the synthesis of abiotic stress response-related secondary metabolites.

## Introduction

Radiata pine (*Pinus radiata* D. Don) is a species native to the Central Coast of California and Mexico. Due to its rapid, versatile growth and the desirable quality of its pulp and lumber, it is suitable for a great range of uses and it is the most widely planted pine in the world (Escandón et al., 2017), extending from New Zealand to Chile, Australia, South Africa, and Spain. Owing to its great economic relevance, major emphasis has been placed during the past 50 years on the development of breeding programs (Espinel et al., 1995; Dungey et al., 2009). However, expected future climate change conditions will seriously threaten the survival and productivity of radiata pine plantations because of increased wind damage, more severe fungal infections, and a high frequency of fierce drought periods (Booth and McMurtrie, 1988; Spinoni et al., 2018). Accordingly, plant capacity to cope with environmental variations will be a key factor for the success of the plantations, with drought being one of the major limiting factors (Mena-Petite et al., 2004).

Somatic embryogenesis (SE) is an effective method for the large-scale propagation of “elite” plants and it can be combined with other technologies such as cryopreservation and selection of clones in field tests (Park, 2002). Moreover, it has been widely used as a model system for understanding the physiological and biochemical events occurring both during plant embryo development and in response to different abiotic stresses (Eliášová et al., 2017). SE in *Pinus radiata* was reported for the first time in by Smith (1997) and in recent years several studies have been made seeking to overcome various obstacles in the process (Hargreaves et al., 2002; Montalbán et al., 2010, 2012, 2015; Montalbán and Moncaleán, 2017, 2018).

Epigenetic variation is thought to be one of the main evolutionary strategies that endow plants with high phenotypic plasticity and an adaptive capacity to changing environments (Boyko and Kovalchuk, 2008; Bräutigam et al., 2013; García-Mendiguren et al., 2017) and clear examples, such as the phenomenon of what is traditionally called plant hardening (Villar-Salvador et al., 2004; Conrath et al., 2015) support this idea. In the so-called deficit irrigation watering strategy, long-term drought tolerance can be induced by transient or partial drying, which could similarly be explained in terms of epigenetic mechanisms (Davies et al., 2010). These mechanisms do not involve mutation or other types of genome-related alterations. They are, by contrast, an active part in the formation of an epigenetic memory that results in modified metabolic and hormonal profiles. These modifications are responsible for the abovementioned long-lasting response which is observed in plants after an initial induction. Under different kinds of stress, which usually lead to the formation of oxygen radicals and osmotic imbalances, plants adjust many of their biochemical pathways and accumulate elevated amounts of metabolites. In many forest species, organic solutes, specifically soluble carbohydrates and free amino acids, are the principal compounds involved in this process (Patakas et al., 2002).

It has been reported that in some tree species such as Norway spruce (*Picea abies*), the temperature during zygotic embryogenesis appears to establish an adaptive epigenetic memory which may regulate the bud phenology and thus, the cold acclimation of the progeny, making this species well adapted to a large range of climatic and geographic areas (Kvaalen and Johnsen, 2008). Colder than normal conditions during zygotic embryogenesis result in plants presenting an early bud set and cold hardiness in autumn along with an early dehardening and bud flushing during spring, whilst higher temperatures delay these processes. Besides, it has been observed that the alterations are durable in progeny with identical genetic background (Johnsen et al., 2005; Yakovlev et al., 2011). In other long-lived plants such as Scots pine or Maritime pine it has been proved that maternal environmental conditions have a direct effect on the performance and phenotype of the offspring (Dormling and Johnsen, 1992; Zas et al., 2013). This “natural hardening” process suggests that embryogenesis may be a critical stage in modulating future plant behavior, thus becoming, in combination with an appropriate propagation system, a valuable strategy for the production of “elite” plants adapted to different environmental conditions.

Following the experiments performed previously in our laboratory (García-Mendiguren et al., 2017), in this work we hypothesize that the application of high temperatures, which are known to reduce water availability (García-Mendiguren et al., 2016b), during the initiation stage of SE, may induce an epigenetic mark that could result in the formation of plantlets with a different capacity to tolerate drought stress through the accumulation of specific metabolites. SE at different induction temperatures has already been studied at a genetic and a transcriptional level for Norway spruce to explain the climatic adaptation of this species (Yakovlev et al., 2011, 2016); moreover, previous studies carried out in our laboratory suggest that the temperature at the initial stages of SE strongly influences the success of the subsequent steps (García-Mendiguren et al., 2016b; Pereira et al., 2016), even determining the protein profile of the obtained somatic embryos (ses) (García-Mendiguren et al., 2016a). Some authors also emphasize the idea that stress could be an essential force to switch on the machinery required for SE (Fehér et al., 2003). In further studies the same authors pointed out that temperature exerts a selective pressure during the initial SE stages that result in lower rates of initiation but an improved yield for the forthcoming steps (Fehér, 2015).

Taking into account the abovementioned information, the aim of this work was to evaluate the effect of high temperatures (30, 40, 50, and 60°C) applied during the initial steps of SE in terms of initiation, proliferation and maturation rates, as well as the quantity and quality of the ses obtained. In the same way, we have tried to elucidate if the stresses applied provoke any type of morphological or ultrastructural alteration in the embryogenic cultures at cellular level. Finally, an analysis of carbohydrate, amino acid and protein profiles has been carried out at initial steps of SE to analyze their behavior under different temperatures.

## Materials and Methods

### Plant Material and Initiation Experiment

One year-old green female cones of *P. radiata* D.Don were collected in July 2016 and 2017 from four different open-pollinated trees (7, 12, 14, and 42) in a seed orchard established by Neiker-Tecnalia in Deba (Spain; latitude: 43°16′59″N, longitude: 2°17′59″W, elevation: 50 m). Cones were wrapped in filter paper and stored at 4°C for one month following Montalbán et al. (2015). They were then surface-sterilized with 70% (v/v) ethanol, split into quarters and all the immature seeds were extracted and surface sterilized following Montalbán et al. (2012). Seed coats were removed and intact megagametophytes were excised out aseptically and placed horizontally onto EDM initiation medium (Walter et al., 2005) supplemented with 3.5 gL^-1^ gellan gum (Gelrite^®^; Becton Dickinson).

#### Experiment 1

For this experiment, cones collected in 2016 were utilized and the assessment of temperatures and the duration of the treatments were based on preliminary studies carried out in our laboratory. In this case, the treatments were as follows: 23°C (8 weeks, control), 30°C (4 weeks), 40°C (4 days), and 50°C (5 min). The Petri dishes with medium were warmed up to the desired temperatures prior to placing the explants on the surface of the medium. Once the different treatments had finished, all the megagametophytes were kept at 23°C for 8 weeks in darkness. In summary, the experiment included four different treatments and four seed families, comprising a total of 960 megagametophytes.

After 8 weeks on the initiation medium, initiation rates were calculated and the emerging embryonal masses (EMs) bigger than 3–5 mm in diameter were separated from the megagametophytes and subcultured to a fresh EDM initiation medium. After 14 days, the EMs were subcultured onto an EDM proliferation medium (the same composition as the initiation medium but a 4.5 gL^-1^ of gellan gum (Gelrite^®^; Becton Dickinson) every 2 weeks until maturation, as described by Montalbán et al. (2010). Following four periods of subculturing, actively growing EMs were recorded as established cell lines (ECLs), and the percentage of proliferating lines respect to the EMs initiated was calculated. For maturation, 8 replicates were prepared per embryogenic cell line (ECL) and each replicate contained 80 mg of EM. Proliferation and maturation were both conducted in darkness at 23°C. After 12–16 weeks, the maturation success was evaluated and the number of ses per gram of EM were calculated.

#### Experiment 2

In this second experiment, cones from 2017 were used and, based on the result of Experiment 1, higher temperatures and shorter time periods were tested. Therefore, the megagametophytes were cultured at 23°C (8 weeks), 40°C (4 h), 50°C (30 min), and 60°C (5 min). The following steps were carried out in the same way as in Experiment 1 with slight differences. In this case, just before their placement onto an EDM proliferation medium, a small part of all the proliferating ECLs was frozen in liquid nitrogen and stored at -80°C. In addition, after assessing the maturation success, the morphology of the obtained ses was studied. There were 10 somatic embryos per each of the four selected ECLs per temperature treatment (4). In summary, 160 somatic embryos were analyzed. For this purpose, two measurements were made using a Leica DMS 1000 microscope and the LAS V4.12 (Leica Application Suite) software: the total length of the ses and the width, measured just under the cotyledons.

### Micromorphological Study

Light microscopy analyses were carried out with EMs which had been frozen after the first proliferation subculture following the procedure described by Fraga et al. (2015). Three ECLs were used per treatment, comprising a total of 12 ECLs. Samples of approximately 3–5 mm in diameter were collected and fixed in formaldehyde (2.5%) in 0.1 M sodium phosphate buffer (pH 7.2) overnight at 4°C. Subsequently, the material was washed twice for 15 min in buffer without fixative and then dehydrated in an increasing series of ethanol aqueous solutions (30–100%), comprising a total of six different solutions, each being applied for 30 min. Then, the samples were infiltrated with Historesin (Leica Historesin, Heidelberg, Germany) and sections of 5 μm were obtained using a rotatory microtome (Slee Technik). After adhesion to histological slides, sections were stained with 1% toluidine blue in an aqueous solution of 1% Borax pH 9. Samples were analyzed and photographed using an Olympus BX 40 microscope equipped with a computer-controlled Olympus DP 71 digital camera.

### Ultrastructural Analysis

The same 12 ECLs were subjected to transmission electron microscopy (TEM). To this end, samples of 3–5 mm in diameter were fixed in 2.5% v/v glutataldehyde in 0.1% w/v sodium cacodylate buffer and 0.6% w/v sucrose overnight. After five washing steps, each of 20 min, with decreasing concentrations of glucose in 0.1% w/v sodium cacodylate buffer, the samples were post-fixed using 1% osmium tetroxide and 0.1 M sodium cacodylate for 4 h, washed again three times with the same concentration of sodium cacodylate and dehydrated in an increasing series of acetone aqueous solutions (30–100%), following the same procedure described above for light microscopy assays. Then, the material was embedded in Spurr’s resin (Spurr, 1969) and ultrathin sections (60 nm) were collected and stained on grinds using aqueous uranyl acetate followed by lead citrate (Reynolds, 1963). The samples were then examined under TEM JEM 1011 (JEOL Ltd., Tokyo, Japan, at 80 kV).

### Metabolites Analysis

Metabolite analysis was performed with the same frozen 12 ECLs used for the micromorphological and ultrastructural analyses. Frozen samples were powdered and diluted 1/12 in ethanol 80% as described by Carillo et al. (2012) with slight modifications. This solution, which will be referred to below as main extract, was used to determine amino acid, total proteins, and carbohydrates.

#### Amino Acid Content Determination

For primary amino acids (alanine, arginine, asparagine, aspartate, ethanolamine, phenylalanine, GABA, glycine, glutamate, glutamine, isoleucine, histidine, leucine, lysine, methionine, ornithine, proline, serine, tyrosine, threonine, tryptophane and valine), distilled water was added to the main extract so that the final concentration was ethanol: water in a ratio of 2:3 (v/v). Following this, all samples were centrifuged for 30 s at 14.000 rpm and diluted by ½ in ethanol 40%. Amino acids were estimated by HPLC after pre-column derivatization by *o*-phthaldialdehyde (OPA) according to Carillo et al. (2011b) using a reverse phase 5 μm ZORBAX Eclipse Plus C18 (250 × 4.6 mm internal diameter; Agilent Technologies). The extract was injected into the column and eluted at a flow rate of 0.85 ml/min at 27° with a discontinuous gradient. Solvent A was a mixture of 50 mM sodium acetate, 20% (v/v) methanol and 3% (v/v) tetrahydrofuran. Solvent B was pure methanol. The amino acid-OPA derivatives were detected by their fluorescence with excitation at 330 nm and emission at 450 nm.

Proline was determined following the procedure of Bates et al. (1973) and described in Carillo et al. (2011a) with slight modifications. Briefly, aliquots of ethanol (containing 2/3 water) previously prepared for amino acid analysis were used. Three replicates of 40 μl were taken from each sample and 80 μl of a reaction mix prepared with ninhydrin 1% (w/v) in acetic acid 60% (v/v) and ethanol 20% (v/v) were added. Then, the samples were kept in the heating block at 90°C and mixed for 20 min at 1400 rpm. After cooling at room temperature, centrifugation was carried out at 14000 rpm for 1 min. Following this, 100 μl of each sample were dispensed in a polypropylene microplate and introduced in a Safas Monaco microplate reader for absorbance measurement at 520 nm.

#### Total Protein Content Determination

Total protein content was determined by the method described by Bradford (1976) with bovine serum albumin as standard as described by Augusti et al. (1999).

#### Carbohydrate Content Quantification

Sugars and starch were measured from the main extract according to Pietrini et al. (1999) with slight modifications. After subsequent extraction steps using decreasing concentrations of ethanol, mixing at 80°C for 20 min and centrifugation at 14000 rpm for 5 min, the supernatant was immediately analyzed for soluble sugars (glucose, sucrose, and fructose) or stored at -20°C until analysis. The pellet, containing starch, was re-suspended in KOH 0.1M, heated for 2 h at 94°C and centrifuged for 1 min at 10000 rpm. pH correction (pH 7) was carried out using acetic acid. To dissolve starch, 50 mM of sodium acetate buffer, containing four units of β-amilase and 40 units of amyloglucosidase were added and the mixture was incubated at 30°C for 2 days to allow complete starch hydrolysis. Following this, the samples were centrifuged and the supernatant analyzed for glucose. Soluble sugars, as well as glucose resulting from the hydrolysis of starch, were analyzed enzymatically as described by Jones et al. (1977). Sucrose and fructose were determined by the addition of 2 units of invertase and 1 unit of isomerase, respectively. The assay was performed using a Safas Monaco microplate reader with wavelength fixed at 340 nm and a measurement interval of 1 min.

### Data Collection and Statistical Analysis

Once the initiation and proliferation rates had been calculated in Experiments 1 and 2, a logistic regression was performed to assess the effect of temperature on both parameters. The mother tree was introduced into the model as a block variable to reduce variability, and temperature was considered as the variable factor. Multiple comparisons were based on predictable linear functions of model coefficients, and Tukey’s *post-hoc* test (α = 0.05) was used. *p* values were conveniently adjusted according to the Benjamini and Hochberg (1995) method.

In relation to maturation results, a logistic regression and the corresponding analysis of deviance were conducted to assess the effect of temperature on the number of mature ses per gram of EM. The ECL was included in the model as a random effect with a different variance for each temperature level. The inclusion of the ECL improved the fit and helped to analyze the effect of temperature more accurately.

As for the evaluation of ses morphology, the length and length/width ratio measurements were analyzed following the same procedure described for maturation. A logistic regression including the ECL as a random effect with the corresponding analysis of deviance was carried out to evaluate the effect of temperature on both measurements. In this case a different variance was considered for each ECL level. Multiple comparisons were based on a Tukey *post-hoc* test (α = 0.05).

Finally, for the data obtained from metabolites analysis, a linear mixed effect model with the ECL as a random effect and the corresponding analysis of variance were performed to assess the effect of temperature on the amount of each metabolite in logarithmic scale. Multiple comparisons were based on Tukey *post hoc* tests.

## Results

### Initiation Experiment

#### Experiment 1

It should be mentioned that megagametophytes subjected to 40°C for 4 days failed to initiate, so no results could be obtained for proliferation and maturation (Table 1). As for theother three treatments, statistically significant differences were found at initiation percentages between treatments 23 and 30°C, and 50°C and 30°C (*p* < 0.05) (Table 2). 30°C was the treatment with the lowest initiation rate, compared with the values obtained for 50 and 23°C, which did not show significant differences between them (Table 1). Concerning proliferation, similar results were observed but differences were significant between all the treatments (*p* < 0.05) (Table 2). Once again, treatment 30°C presented the lowest values and the highest proliferation rates were obtained when treatment at 50°C was applied, followed by treatment 23°C (Table 1).

**Table 1:** Embryonal mass initiation and proliferation (%) in *Pinus radiata* megagametophytes cultured at different temperatures in EDM for Experiment 1 and Experiment 2.

Experiment 1			Experiment 2		
Treatment	Initiation %	Proliferation %	Treatment	Initiation %	Proliferation %
23°C, 8 weeks	56.9 ± 3.12^a^	29.9 ± 2.89^2^	23°C, 8 weeks	12 ± 2.14^a^	10.6 ± 2.12^1^
30°C, 4 weeks	23.3 ± 3.20^b^	6.2 ± 1.86^3^	40°C, 4 h	9.6 ± 2.24^a^	6.7 ± 1.64^1^
40°C, 4 days	0 ± 0	0 ± 0	50°C, 30 min	5.9 ± 1.59^a^	4.1 ± 1.26^1^
50°C, 5 min	54.7 ± 3.44^a^	37.2 ± 3.46^1^	60°C, 5 min	10.8 ± 2.35^a^	8.5 ± 2.10^1^

*Data are presented as mean values ± SE. Significant differences at *p* < 0.05 are indicated by different letters for initiation and by different numbers for proliferation.*

**Table 2:** Analysis of deviance of the logistic regression for initiation and proliferation (%) of *P. radiata* embryonal masses in Experiment 1 and Experiment 2 according to temperature.

Experiment 1				Experiment 2			
Source	df	X^2^ test	*p* value	Source	df	X^2^ test	*p* value
**Initiation**				**Initiation**			
Temperature (T)	2	112.845	<2.2 × 10^-16^	Temperature (T)	3	5.8749	0.11786
**Proliferation**				**Proliferation**			
Temperature (T)	2	124.13	<2.2 × 10^-16^	Temperature (T)	3	7.7246	0.05206

Maturation showed no variations with respect to temperature, as 59 out of the 64 ECLs (92.2%) accomplished maturation. No significant differences were found for the number of ses produced per gram of EM either (*p* < 0.05) (Supplementary Table 1). However, it is noteworthy that ECLs derived from treatments 50 and 30°C produced the highest number of ses, compared with those from treatment 23°C (Table 3).

**Table 3:** Number of somatic embryos per gram of embryonal mass obtained under different initiation temperatures in Experiment 1 and Experiment 2.

Experiment 1		Experiment 2	
Treatment	ses/g EM	Treatment	ses/g EM
23°C, 8 weeks	334.55 ± 27.13	23°C, 8 weeks	207.12 ± 32.74
30°C, 4 weeks	520.88 ± 63.1	40°C, 4 h	283.68 ± 72.22
40°C, 4 days	–	50°C, 30 min	256.25 ± 40.3
50°C, 5 min	564.71 ± 56.96	60°C, 5 min	216.32 ± 22.33

*Data are presented as mean values ± SE.*

#### Experiment 2

The initiation percentage did not show statistically significant differences between the assayed temperatures (*p* < 0.05) (Table 2). The same results were obtained for proliferation, despite the fact that the differences observed for proliferation percentages are bordering on being statistically significant (*p* = 0.05206). However, it is remarkable that both initiation and proliferation rates showed the same decreasing pattern when high temperatures were applied, with 23°C being the treatment with the highest initiation and proliferation percentages and 50°C the one with the lowest percentages. With respect to the other two treatments (40 and 60°C), they displayed an intermediate behavior, with the results obtained for 60°C more similar to the ones for 23°C (Table 1).

Regarding maturation, 23 out of the 24 ECLs subjected to this process (95.8%) produced ses, which clearly shows that temperature had no effect on the maturation capacity of the ECLs. As for the number of ses per gram of EM, no significant differences were found between the different temperatures (*p* < 0.05) (Supplementary Table 1). All average values were around 200–300 ses per gram of EM, with 23°C being the treatment with the lowest and the highest value corresponding to 40°C (Table 3).

**FIGURE 1:**
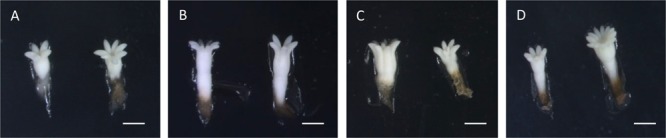
Somatic embryos produced under different initiation temperatures: 23°C, 8 weeks **(A)**, bar = 1 mm; 40°C, 4 h **(B)**, bar = 1.4 mm; 50°C, 30 min **(C)**, bar = 2.2 mm; and 60°C, 5 min **(D)**, bar = 1.7 mm. Notice the different morphologies between temperatures. Embryos from temperatures 40 and 60°C showing an elongated shape, while embryos from temperature 23°C are short and embryos from 50°C are big but barrel-shaped.

Nevertheless, statistically significant differences were found for the length and the length/width ratio of the obtained ses (*p* < 0.05). Ses derived from ECLs initiated at 50°C were significantly longer (3.76 ± 0.1 mm) than those from control treatments (2.34 ± 0.16 mm). Ses derived from treatments 40 and 60°C presented intermediate values (3.08 ± 0.13 and 3.05 ± 0.12 mm, respectively). With respect to length/width ratio, ses from initiation temperatures 23 and 50°C showed the lowest values (2.408 ± 0.06 and 2.435 ± 0.1), denoting a barrel-like shape, especially those corresponding to treatment 50°C. On the other hand, ses from treatments 40 and 60°C resulted in the highest values (2.872 ± 0.09 and 2.703 ± 0.09), denoting a more elongated shape (Figure 1).

### Micromorphological Study

Micromorphological analyses revealed the presence of three cell types in the embryogenic cultures:

1. Embryogenic cells (ECs), which were small, round-shaped and had a dense cytoplasm, with large central nuclei (N), characteristics, all these being typical of meristematic cells.
2. Suspensor cells (SCs), which were elongated, according to their degree of vacuolation, and which had neither N nor organelles (in some cases a few degraded organelles were detected).
3. Embryonal tube-like cells (TLCs), seemed to be in transition between ECs and SCs as they share characteristics of both cell types. They had a big central N and a dense cytoplasm, but they showed an increase in length and in the number of vacuoles (V).

All these cells were attached to each other forming aggregates called proembryogenic masses (PEMs) and in all cases three developmental and organizational stages of PEMs were observed: PEMI, PEMII, and PEMIII. PEMI were characterized by the presence of small groups of ECs linked to one or two SCs, while PEMII and PEMIII consisted of a higher number of ECs and increased numbers of SCs, forming large cell clusters in the case of PEMIII. However, characteristics in terms of cell organization, polarization and structure differed significantly between the treatments.

**FIGURE 2:**
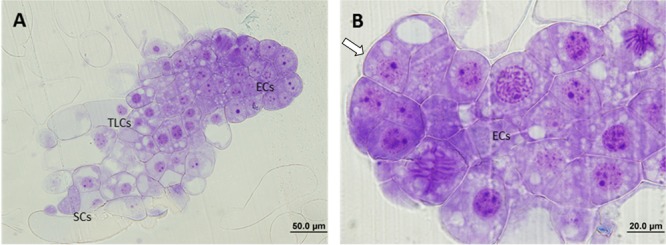
Light microscopy and histochemical analyses of proembryogenic masses (PEMs) of *Pinus radiata* induced at 23°C. **(A)** PEMIII showing clear polarization and a well divided structure: compact embryogenic area formed by embryogenic cells (ECs),transition region formed by tube-like cells (TLCs) and suspensor cells (SCs). **(B)** Detail of an embryogenic mass with high mitotic activity and thick layer of polysaccharides (arrow).

In the case of the ECLs initiated at 23°C, all three stages of PEMs could be observed in the analyzed samples and polarity was evident for all of them, especially for PEMIII. These structures showed a clear organization, divided into three regions, with an embryogenic dense mass composed of ECs at the top, followed by a transitional region formed by TCLs and a suspensor region at the end (Figure 2A). The cells at the embryogenic areas presented a smooth surface with a thick layer of polysaccharides and a high metabolic and mitotic activity (Figure 2B). SCs were long and most of them remained attached to the structure.

**FIGURE 3:**
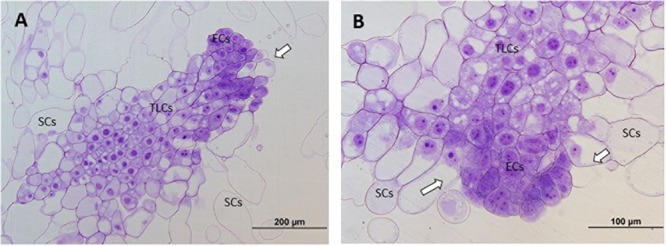
Light microscopy and histochemical analyses of PEMs of *P. radiata* induced at 40°C during 4 h. Clear examples of PEMIIIs starting to loose polarization with SCs arising directly from embryonal areas **(A)** or close to them **(B)** (arrows). Note the increase of vacuolation and the decrease of mitotic activity in ECs.

At 40°C, however, polarized structures were not always present. Even though a small grade of polarization was observed in some cases, most of the PEMs could not be divided into three regions. The reason for this was the appearance of SCs near embryogenic areas, without a clear and thick transition region dividing them, or just the formation of SCs directly from embryogenic areas, disturbing the cell cluster. It is also noticable that ECs presented a decrease in mitotic activity and an increase in vacuolation (Figure 3).

**FIGURE 4:**
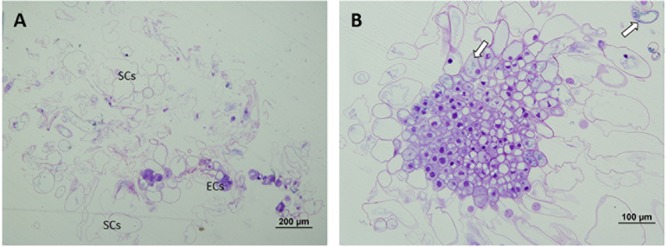
Light microscopy and histochemical analyses of PEMs of *P. radiata* induced at 50°C for 30 min. **(A)** General view of a sample presenting supernumerary SCs detached from small embryonal areas. Notice the great number of degraded SCs. **(B)** Embryonal area that has completely lost polarization and presents symptoms of PCD. Cells have a high rate of vacuolation, start to elongate to form SCs and heterochromatin becomes more visible. Small phenolic grains start to appear (arrows).

At 50°C, polarization was non-existent. No cell organization could be observed. SCs arose directly from embryonal areas and most of them appeared detached from PEMs, presenting an advanced state of degradation in many cases. The number of SCs was also much higher than in the other treatments and embryonal areas were significantly smaller in this case, creating an unbalanced proportion between embryonal areas and suspensors (Figure 4A). Embryonal areas showed symptoms of early vacuolar programmed cell death (PCD) and loss of embryogenic state, such as an increase of vacuolation and a decrease of mitotic activity, as described by Smertenko and Bozhkov (2014). As well as this, in some of the cells, small blue-stained spots were detected attached to the inner faces of tonoplasts, highlighting the presence of phenolic compounds (Figure 4B).

**FIGURE 5:**
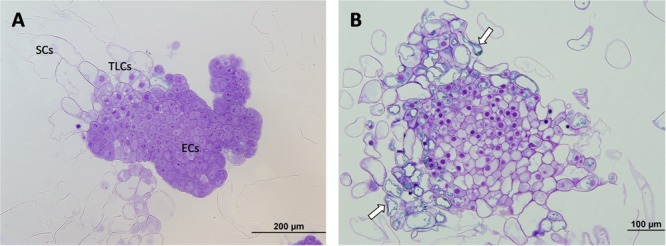
Light microscopy and histochemical analyses of PEMs of *P. radiata* induced at 60°C for 5 min. **(A)** PEMIII formed by a huge cluster of compact ECs resembling the first steps of somatic embryo development. **(B)** Detail of a group of ECs that have lost their embryogenic state and are developing into SCs. Note the absence of polarization, the non-organized formation of SCs and the presence of large amounts of phenolic compounds attached to the inner layer of vacuole membranes, stained in blue (arrows).

Finally, for ECLs initiated at 60°C, both polarized and non-polarized areas were detected. In the case of non-polarized areas, the same degeneration pattern was observed as in samples initiated at 50°C, as well as the accumulation of abovementioned phenolic compounds (Figure 5B). In the case of polarized areas however, embryonal areas were even bigger than in the control samples and were tightly attached to each other, forming more developed structures that resembled early globular somatic embryos (Figure 5A).

#### Ultrastructural Analysis

**FIGURE 6:**
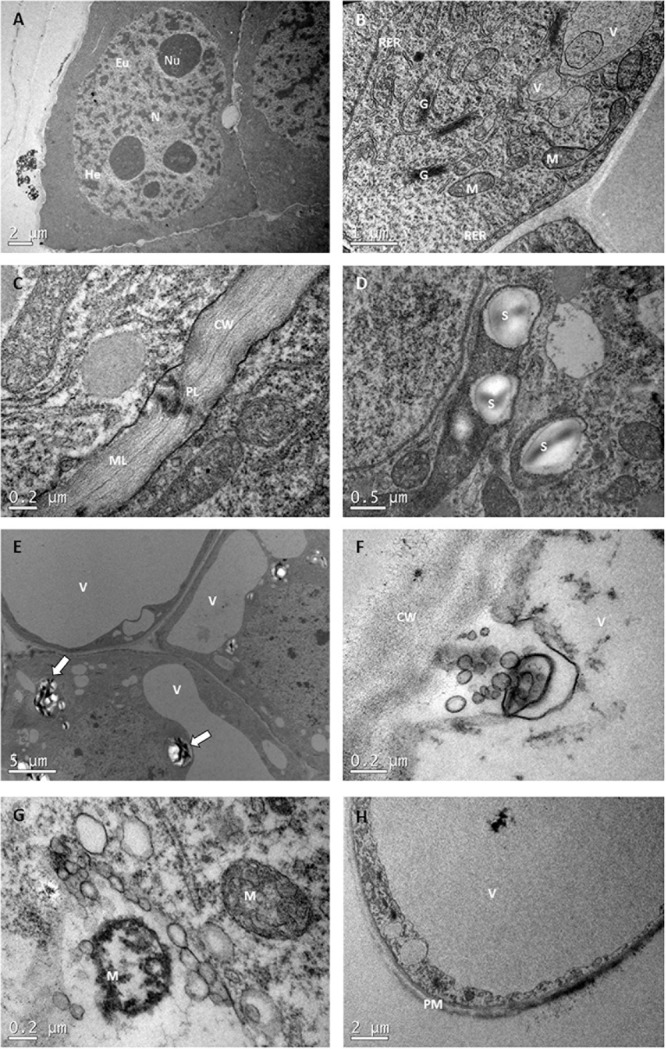
Transmission electron microscopy images of *P. radiata* ECs, TLCs, and SCs. **(A)** EC with huge central nuclei (N) presenting four nucleolus (Nu), heterochromatin (He) and euchromatin (Eu). Notice the high nuclei: cytoplasm ratio and the dense cytoplasm. **(B)** Detail of the EC cytoplasm with rough endoplasmic reticulum (RER), Golgi bodies (G), small vacuoles (V) and mitochondria (M). Observe the “sickle-like mitochondria” close to the plasma membrane (arrow). **(C)** Detail of the cell wall (CW), with well-organized fibers, visible middle lamella (ML) and some plasmodesmata (PL). **(D)** Image of plastids containing starch grains (S). **(E)** TLCs with big vacuoles and amyloplasts (arrows). **(F,G)** showing clear examples of cell dismantling and organelle dergradation. Notice the disorganization of cell wall fibers. **(H)** Image of an SC with a huge central vacuole taking up most of the cell volume. The cytoplasm, with a few quite degenerated organelles occupies a narrow layer confined between tonoplast and plasma membrane (PM).

As a general trend, TEM enabled observations of three cell types: ECs, TLCs and SCs. The ECs morphology, as briefly described previously, was characterized by a dense cytoplasm with a large, prominent central N, high ratio nuclei: cytoplasm, and distinguishable heterochromatin and euchromatin regions. Big nucleoli and intact nuclear envelope could also be observed (Figure 6A). In the cytoplasm many organelles such as, mitochondria (M), rough endoplasmic reticulum, Golgi bodies (G), small V and plastids, both with and without, starch grains (S) were present (Figures 6B,D). The cell wall was thin with a few plasmodesmata and a visible middle lamella (Figure 6C).

Ultrastructural characterization of TCs and SCs, however, showed that these cells were undergoing different degrees of cellular disassembly as compared to cellular organization of ECs (Filonova et al., 2000). TLCs differed from ECs by being more elongated, with a thicker and less organized cell wall and a higher number and larger V. At the same time, many leucoplasts developed into amyloplasts, presenting huge S, and the number of G increased significantly, which resulted in the formation of largle amounts of vesicles (Figure 6E). In SCs, engulfment of those vesicles and portions of cytoplasm by V gave rise to the formation of a big autolytic vacuole, that together with plastolysome-like structures (portions of cytoplasm surrounded by one or several double membranes arising from a plastid-like leucoplast) started to progressively destroy the cytosol and organelles, taking up the majority of the cell volume until its collapse and the formation of a hollow-walled corpse (Figures 6F–H).

**FIGURE 7:**
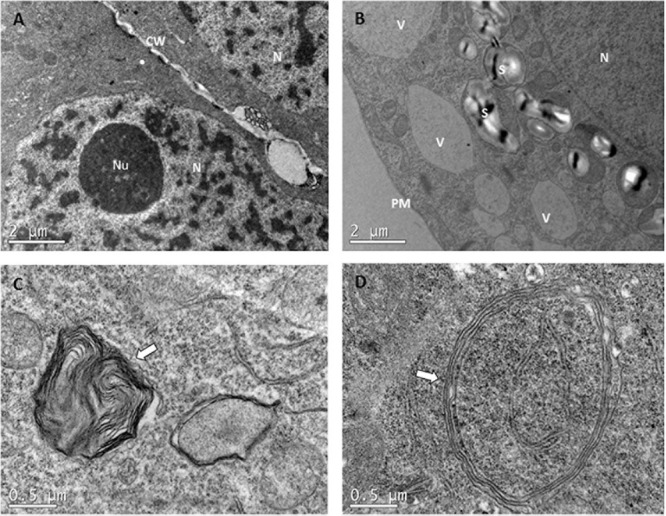
Transmission electron microscopy images comparing ECs that came from PEMs initiated at different temperatures. **(A)** EC from 23°C treatment. Observe the presence of a dense cytoplasm, with no or just a few small provacuoles. No starch grains can be detected. **(B)** EC from 40°C for 4 h with a lot of vacuoles, bigger than in the control treatment, and many plastids containing starch grains surrounding the nuclei. Both symptoms of PCD. **(C,D)** are details of the cytoplasm of ECs from 50°C for 30 min, showing whorls of cytoplasmic membranes and plastolysome-like structures, respectively.

Despite these general characteristics of each cell type, some differences were detected among treatments, supporting the results obtained from light microscopy assays. As a general trend, ECs from control samples (23°C), presented a dense cytoplasm with no or just a few V. Neither amyloplasts nor plastids with small grains of starch could be detected in these samples (Figure 7A). In the case of samples subjected to high temperatures, however, G became more numerous, the number of small V increased noticeably, and the presence of a big S around the N was noticeable (Figure 7B). Even though small amounts of phenolic compounds could be detected in the inner face of tonoplasts, as described before in light microscopy experiments, no clear differences were seen among treatments in TEM analysis. For ECLs initiated at 50 and 60°C, big plastolysome-like structures (Figure 7D) and the formation of whorls by cytoplasmic membranes (Figure 7C) were identified. No clear differences among the treatments were observed for tube-like cells and SCs.

TEM analysis also revealed different types of M morphologies. Besides the typical cylindrical shaped with well-organized cristae morphology, we observed swollen M, mainly in cells undergoing PCD such as tube-like cells or SCs, and “sickle-like M” (Figure 6B).

### Metabolites Analysis

Among all the metabolites analyzed, soluble sugars, starch, total protein, and the amino acids alanine, arginine, asparagine, aspartate, ethanolamine, phenylalanine, GABA, glycine, glutamate, glutamine, lysine, methionine, ornithine, proline, serine, threonine, tryptophan, and valine presented no significant differences (Tables 4, 5 and Supplementary Table 2). However, the applied stress resulted in significantly different contents of leucine, isoleucine, tyrosine and histidine (Table 5 and Supplementary Table 2).

**Table 4:** Carbohydrates (μmol/g FW) and soluble proteins (mg/g FW) in *P. radiata* embryonal masses under different initiation temperatures (23°C, 8 weeks; 40°C, 4 h; 50°C, 30 min; 60°C, 5 min).

	Treatment (°C)			
Carbohydrates (μmol/g FW)	23	40	50	60
Starch	6.4 ± 2.681^a^	3.36 ± 0.89^a^	5.77 ± 3.88^a^	5.32 ± 2.61^a^
Glucose	37.17 ± 2.91^a^	33.53 ± 2.28^a^	31.85 ± 4.65^a^	33.13 ± 1.7^a^
Fructose	40.48 ± 3.98^a^	38.86 ± 1.48^a^	44.77 ± 4.24^a^	41.63 ± 1.75^a^
Sucrose	7.73 ± 0.37^a^	6.74 ± 0.12^a^	7 ± 0.6^a^	7.17 ± 0.28^a^
**Proteins (mg/g FW)**				
Total protein	1.06 ± 0.16^a^	1.02 ± 0.08^a^	0.99 ± 0.03^a^	1.05 ± 0.12^a^

*Data are presented as mean values ± SE. Significant differences at *p* < 0.05 are indicated by different letters.*

**Table 5:** Free amino acids (μmol/g FW) in *P. radiata* embryonal masses under different initiation temperatures (23°C, 8 weeks; 40°C, 4 h; 50°C, 30 min; 60°C, 5 min).

	Treatment (°C)			
Amino acids (μmol/g FW)	23	40	50	60
Alanine	8.33 ± 3.83^a^	7.6 ± 3.42^a^	7.38 ± 1.45^a^	6.09 ± 1.49^a^
Arginine	1.19 ± 0.5^a^	0.48 ± 0.18^a^	0.62 ± 0.24^a^	1.51 ± 0.68^a^
Asparagine	16.6 ± 4.11^a^	11.1 ± 3.38^a^	12.23 ± 2.11^a^	13.79 ± 3.48^a^
Aspartate	0.12 ± 0.02^a^	0.08 ± 0.02^a^	0.09 ± 0.02^a^	0.12 ± 0.01^a^
Ethanolamine	0.19 ± 0.01^a^	0.3 ± 0.12^a^	0.15 ± 0.02^a^	0.29 ± 0.09^a^
Phenylalanine	0.1 ± 0.01^a^	0.12 ± 0.01^a^	0.1 ± 0^a^	0.11 ± 0.01^a^
GABA	0.06 ± 0^a^	0.07 ± 0.02^a^	0.07 ± 0^a^	0.08 ± 0^a^
Glycine	1.02 ± 0.08^a^	1.1 ± 0.05^a^	1.06 ± 0.11^a^	0.97 ± 0.14^a^
Glutamate	0.18 ± 0.02^a^	0.29 ± 0.1^a^	0.17 ± 0.03^a^	0.21 ± 0.04^a^
Glutamine	6.89 ± 0.73^a^	8.51 ± 1.44^a^	10.63 ± 3.37^a^	7.46 ± 3.89^a^
Isoleucine	0.15 ± 0.01^c^	0.2 ± 0.01^a^	0.16 ± 0.01^bc^	0.19 ± 0.02^ab^
Histidine	1.33 ± 0.21^ab^	2.16 ± 0.59^a^	1.26 ± 0.13^b^	1.48 ± 0.14^ab^
Leucine	0.16 ± 0.02^a^	0.19 ± 0.01^a^	0.1 ± 0.01^b^	0.2 ± 0.02^a^
Lysine	0.22 ± 0^a^	0.24 ± 0.02^a^	0.22 ± 0^a^	0.27 ± 0.02^a^
Methionine	0.06 ± 0^a^	0.06 ± 0^a^	0.07 ± 0.01^a^	0.07 ± 0.01^a^
Ornithine	0.25 ± 0^a^	0.24 ± 0.02^a^	0.25 ± 0.01^a^	0.24 ± 0.03^a^
Proline	2.32 ± 0.53^a^	1.42 ± 0.61^ab^	1.88 ± 1.09^ab^	0.86 ± 0.12^b^
Serine	0.49 ± 0.08^a^	0.54 ± 0.07^a^	0.51 ± 0.17^a^	0.68 ± 0.21^a^
Tyrosine	0.13 ± 0.01^b^	0.25 ± 0.03^a^	0.24 ± 0.02^a^	0.21 ± 0.03^a^
Threonine	0.48 ± 0.06^a^	0.41 ± 0.03^a^	0.71 ± 0.21^a^	0.5 ± 0.2^a^
Tryptophane	0.12 ± 0.01^a^	0.16 ± 0.04^a^	0.12 ± 0.01^a^	0.15 ± 0.03^a^
Valine	0.24 ± 0.05^ab^	0.34 ± 0.07^a^	0.18 ± 0.02^b^	0.28 ± 0.03^a^

*Data are presented as mean values ± SE. Significant differences at *p* < 0.05 are indicated by different letters.*

**FIGURE 8:**
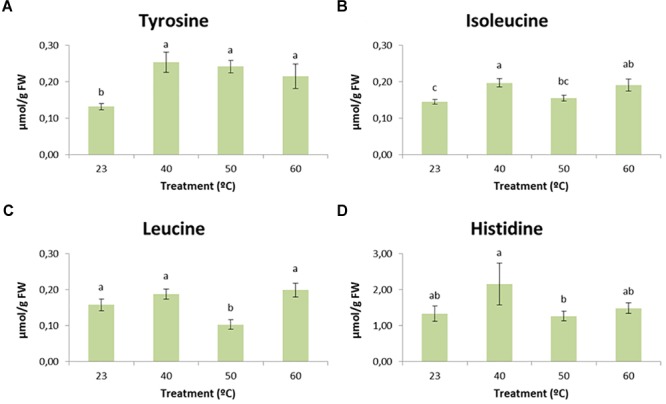
The effect of temperature (23°C, 8 weeks; 40°C, 4 h; 50°C, 30 min; 60°C, 5 min) on tyrosine **(A)**, isoleucine **(B)**, leucine **(C)**, and histidine **(D)** content of *P. radiata* embryonal masses. Data are presented as mean values ± SE. Significant differences at *p* < 0.05 are indicated by different letters.

Regarding these differences, diverse patterns could be observed. In the case of tyrosine (Figure 8A), cell lines initiated at 23°C presented significantly (*p* < 0.05) lower amounts of this amino acid when compared to lines initiated at higher temperatures, showing a clear accumulation when embryogenic masses were initiated under high temperatures. With respect to isoleucine (Figure 8B), lines initiated at 40 and 60°C showed significantly higher values than those initiated at control temperature. Leucine, for its part (Figure 8C), showed significantly (*p* < 0.05) lower amounts in the case of lines initiated at 50°C when compared to those initiated at 23, 40 and 60°C. Despite not being significantly different from the control temperature, leucine followed the same pattern described for isoleucine as the greatest amounts of this amino acid were found in lines subjected to 40 and 60°C. Finally, statistically significant (*p* < 0.05) differences were found for the levels of histidine between treatments 40 and 50°C (Figure 8D). Lines initiated at 50°C presented the lowest levels of histidine, whereas those subjected to 40°C accumulated the highest ones. Application of the control treatment and 60°C resulted in intermediate values, but following once again the tendency described above for leucine and isoleucine.

## Discussion

Somatic embryogenesis is a complex network of genetic, physiological and developmental processes that are strongly regulated and affected by internal and external factors. Small alterations of these factors could result in a complete failure of SE, especially conditioning critical but at the same time highly challenging steps such as initiation (Santa-Catarina et al., 2012). However, different stress conditions could presumably be beneficial and in some cases almost crucial for SE success; thus, heat stress is one of the significant stresses for microspore embryogenesis induction in *Brassica napus* due to changes in DNA methylation (Li et al., 2016). This, in combination with the idea that conditions at the initiation step have long-term effects, could be used as a tool for improvement by increasing the efficiency of the process or the diversity of the plants produced.

Following the same tendency observed in previous studies (García-Mendiguren et al., 2016b; Pereira et al., 2016, 2017), significant differences in initiation and proliferation rates were found when different temperatures were applied during the initiation of SE in *P. radiata*. García-Mendiguren et al. (2016b) concluded that low temperatures (18 and 23°C) give rise to higher initiation rates when compared to those obtained when it was carried out at 28°C.

In the results obtained in Experiment 1, we observed that high temperatures applied for long periods of time resulted in the total (40°C, 4 days) or partial (30°C, 4 weeks) decrease of both initiation and proliferations rates. Thus, we could refer to these treatments as representing strong stresses. In Experiment 2 the effect was not so clear, maybe because the treatments applied were shorter, but we could also observe a slight decrease in both parameters when temperatures above the standard were applied for the longest periods (40°C, 4 h and 50°C, 30 min). In fact, the differences observed for proliferation at 50°C were on the verge of statistical significance. As a result, we can conclude that when high temperatures are applied small differences in the duration of the treatment can be critical and when that point is exceeded the effects can be detrimental for the first steps of SE, compromising the genetic diversity that could be achieved in standard conditions.

In previous experiments carried out in our laboratory (García-Mendiguren et al., 2016b), the opposite effect was observed for proliferation and maturation phases, where high temperatures resulted in considerably higher proliferation rates and larger number of ses. Despite proliferation following the same pattern as initiation in our study, maturation results were similar to those from the abovementioned study. Although not statistically significant, in both experiments the number of ses obtained per gram of EM was slightly higher when stress was present, increasing the efficiency of the process. These results reinforce the idea postulated by Fehér (2015) in, according to which temperature exerts a selective pressure in the SE initial stages, resulting in an initiation decrease but higher rates for the forthcoming steps. It is worth mentioning that both authors, Fehér and García-Mendiguren, obtained better proliferation rates when stress was applied, which is in contradiction to our results. This suggests that the treatments applied in our experiments could have provoked a more severe effect at cellular level and as a result, had a more long-lasting effect on the EMs.

In contrast to these results, we have the ones obtained when high temperatures were applied for short periods of time, which we could refer to as mild stresses (50°C for 5 min in Experiment 1 and 40°C for 4 h and 60°C for 5 min in Experiment 2). In these cases, the values obtained for initiation and proliferation rates were not statistically different from those obtained at control temperatures, with the exception of 50°C for 5 min in Experiment 1 at proliferation, which were even higher than in control temperatures, dismissing the detrimental effect previously described for high temperatures during long time periods. Surprisingly, the beneficial effect observed during maturation when high temperatures were applied for long time periods was also noticeable for mild stresses.

It is also noticeable that mild stresses had the effect of forming big, well-formed elongated ses, while long-term applied high temperatures gave rise to big but barrel-shaped ses. This kind of morphology is known to be a symptom of low quality embryos (Pullman et al., 2003) and it is usually accompanied by low germination rates, especially with regard to rooting. Thus, even though stress induces the formation of ses, long-lasting stresses provoke negative morphological alterations, while mild stresses enhance the quality of ses. Microscopic observations and analysis of cellular ultrastructure may be able to shed light on this.

Light microscopy analysis revealed the presence of three cell types (ECs, TLCs, and SCs) and three organizational structures called PEMI, PEMII and PEMII, as described for *P. abies* by Filonova et al. (2000). These authors described PEMIII as enlarged clumps of densely cytoplasmic cells loosely attached to each other that do not present polarity, whereas in the case of *P. radiata* the ECs observed formed compact clumps which showed, in most cases, a well-organized structure with a clear polarization. A similar model was described by Steiner et al. (2016) for early somatic embryos of *Araucaria angustifolia*, but no polarization was observed in PEMs, which could be explained in terms of interspecific differences.

In both light microscopy and TEM analysis, high temperatures during early stages of SE resulted in loss of polarity and mitotic activity, and an increase in V, G, plastolysome-like structures, whorls by cytoplasmic membranes and S around the N. These events are usually accompanied by nuclear degradation, which is reflected by the normally round N becoming lobed, with large clusters of nuclear pore complexes and contents of chromatin leaking into the cytoplasm (Filonova et al., 2000). Even though many anucleated SCs were observed, none of the above-mentioned symptoms of nuclear degradations were detected in our analysis.

With all this information, we can conclude that heat shock treatments may trigger PCD of ECs. Interestingly, DaMatta and Ramalho (2006) pointed out that strong high temperature-based stresses are known to activate signaling pathways related to plant PCD. An increased PCD leads to an early loss of embryogenic potential and the formation of supernumerary SCs, which in turn may compromise proliferation and maturation of PEMs. This supernumerary suspensor cell scenario may be caused by a disturbed polar auxin transport, which stimulates division of ECs adjacent to the tube-like cells, as described by Abrahamsson et al. (2012). Similar morphologies were described by Merino et al. (2018) for Norway spruce early somatic embryos leading to abnormal somatic embryos. These authors pointed out that there is a strong relationship among early somatic embryo morphology, its capacity to develop into a mature embryo and the expression of certain transcripts. Up-regulation of EXPB1 and RIC3, transcripts suggested to be involved in the cleavage process during zygotic embryogenesis, resulted in the disintegration of EMs, the presence of highly vacuolated cells in the embryonal clusters and the formation of supernumerary SCs. Down-regulation of CYP78A7 or TT7 on the other hand, caused the formation of embryos with disturbed apical-basal polarization. This information indicates that heat-shock treatments may have provoked alterations in the expression pattern of some gens involved in the correct development of PEMs and thus, in the correct development of future ses.

The idea that the accumulation of S around N is a symptom of loss of embryogenic competence was also described by Morel et al. (2014). These authors noticed that starch accumulation occurs mainly during the first weeks of the maturation process, not during the proliferation phase, in which the glycolytic pathway is enhanced, and this accumulation occurs basically in SCs and in the basal part of the embryos. Embryos accumulating S around the N were linked with the appearance of dead cells in meristematic centers.

All these aspects could, to some extent, explain the results obtained for initiation and proliferation rates. The increase in PCD and the loss of embryogenic competence observed in EMs submitted to heat shock treatments, and especially those initiated at high temperatures during longer periods of time, such as those at 50°C in Experiment 2, may be the reason for the slight decrease in both parameters. Previous studies (Breton et al., 2006; Carneros et al., 2017) suggest that these effects could be more relevant during future stages, especially during maturation, where cellular organization may play a crucial role in the success of embryo formation. However, alterations observed during maturation were not as relevant as those observed during initiation or proliferation. The only remarkable aspect was the formation of barrel-shaped ses when long term stresses were applied, which supports the idea that cellular organization of PEMs, at least in our case, had a major impact on the morphology of the forming ses, rather than on the success of the maturation itself.

It is worth noting that in spite of the cellular dismantling aspects observed at 60°C, light microscopy demonstrated the presence of some bigger and more developed embryogenic areas when compared to control samples, which could explain the improvements observed for embryo production and embryo morphology in the case of mild stress temperatures. This is in line with results of authors that pointed out the idea of temperature based mild stresses having a beneficial effect during production of somatic embryos. Besides, it is known that some stresses induce *de novo* synthesis of ABA and therefore enhance endogenous abscisic acid levels (Dronne et al., 1997), which could explain the formation of structures that resembled earlier globular somatic embryos in 60°C samples.

It is also interesting to take into account the differential accumulation of phenolic compounds observed in light microscopy, especially for treatments 50 and 60°C. Accumulation of this type of molecules is described as playing an essential role in the prevention of oxidative damage caused by different types of stresses (Eliášová et al., 2017; Falahi et al., 2018).

Finally, different mitochondrial morphologies were observed by TEM. These morphologies were described for the first time in plant embryogenic cultures by Fraga et al. (2015). Alterations in mitochondrial structure in response to heat shock treatments have already been reported (Welch and Suhan, 1985). However, the alterations observed in our experiment were homogeneously distributed among all treatments, which suggest that other stress factors may be the reason for such changes. *In vitro* culture represents an unusual combination of stress factors (Zavattieri et al., 2010), so it is difficult to assess whether externally applied stresses are responsible for altered mitochondrial conformations or if *in vitro* culture itself promotes those changes.

With regard to the metabolite analysis, significant differences were found for several metabolites for different temperature treatments. The accumulation of metabolites in response to abiotic stresses, and in particular to osmotic stress, is well documented (Delauney and Verma, 1993). Osmotic stress can be provoked due to different kinds of environmental conditions such as drought, salinity, or flooding. Drought is usually accompanied by other stresses such as heat, and recent studies have shown that both these stresses have overlapping roles (Jia et al., 2017). Heat involves complex mechanisms including multiple protective pathways which seem to be essential for homeostasis preservation (Ahuja et al., 2010).

In this regard, sugars seem to be among the most stress-responsive metabolites, as they play an essential role as osmolytes, stress response signals, and they are responsible for membrane stabilization and oxygen radical scavengers (Moradi et al., 2017; Woodrow et al., 2017). However, no differences were found either for soluble sugars, or for starch in our samples. Microscopic observations highlighted the accumulation of bigger amounts of S in samples subjected to higher temperatures, but this could be more related to PCD events rather than to protective ones. Furthermore, the lack of significant differences for HPLC analysis of these molecules might have been caused by the specific localization of starch, principally in cells starting to undergo PCD. This could all be explained by an unbalanced number of cell types among the treatments. In fact, samples submitted to high temperatures were those with the biggest amount of highly degraded SCs, most of which formed hollow-walled corpses.

Amino acid metabolism may also play an important role in plant stress tolerance through the accumulation of compatible metabolites, intracellular pH regulation, and detoxification of reactive oxygen species. These molecules are also known to be precursors of secondary metabolites (Campalans et al., 1999; Nuccio et al., 1999). Among amino acids, it is proline which has been studied most extensively (Corcuera et al., 2012; De Diego et al., 2015), and apart from the abovementioned functions, it may also be involved in the protection of cell structures and cell proteins (Bandurska et al., 2009). Nevertheless, no differences could be observed for this amino acid study. It is worth mentioning, however, that when metabolites were statistically analyzed, the introduction of the cell line in the model improved the adjustment, suggesting that there is variability that could be explained in terms of genetic background. This genetic background could have a great influence when there is exposure to temperature-based stress treatments. Besides, it is known that the accumulation of osmolytes is dependent on the cell or tissue type, developmental status, the particular environment, and the nature of the stress (Burg and Ferraris, 2008). In this sense, studies carried out with tobacco and *Arabidopsis* concluded that heat stress was not effective to promote proline accumulation (Krasensky and Jonak, 2012).

Despite being the most frequently studied amino acid, proline is not the only one whose accumulation pattern is altered in response to stress. Relative increases in branched-chain amino acids (BCAAs) (leucine, valine, and isoleucine) under drought stress have previously been studied in *Arabidopsis* by Nambara et al. (1998), and many studies conclude that the accumulation of this type of amino acid can be much greater under certain stress conditions than proline accumulation (Shen et al., 1989). It is noteworthy that the accumulation of BCAAs is more common in young tissues and reproductive ones (Singh and Shaner, 1995), where cells have high mitotic and metabolic activity, characteristic of EMs.

Our results suggest a slight accumulation, especially for isoleucine, in treatments that we have described as mild stresses (40°C, 4 h and 60°C, 5 min). It has been proposed that accumulation of BCAAs may serve as substrate for the synthesis of stress-induced proteins and that BCAAs may act as signaling molecules to regulate gene expression (Joshi et al., 2010). We have also observed a decrease in BCAA content when strong stress conditions were applied (50°C for half an hour), especially for leucine. The reason for this could be an increased consumption of this amino acid for the synthesis of enzymes or secondary metabolites that could help to cope with stress (Falahi et al., 2018). Some studies reported a transient increase in BCAAs during stress conditions followed by a rapid decrease once the stress was eliminated. The explanation for this may be that BCAA catabolism leads to the formation of energetic compounds that could be very useful during plant recovery (Taylor et al., 2004).

Differences were also observed for the levels of tyrosine and histidine. The first amino acid presented a clear accumulation in all heat-shock treatments. Some authors, like Falahi et al. (2018), detected the same pattern when plants of *Scrophularia striata* were submitted to water deficit. Tyrosine is a precursor for a wide range of secondary metabolites. Among these metabolites, we can highlight the presence of phenolic compounds, which were detected in light microscopy assays as being highly accumulated in samples initiated under stress conditions. With regard to histidine, the greatest values were observed for the 40°C treatment. In previous studies carried out in *Pinus halepensis* seedlings, histidine showed higher concentrations in drought-tolerant plants (Taïbi et al., 2015).

To summarize, we can state that heat based treatments applied during the initial stages of SE of *P. radiata* had varying effects throughout the process. Long-lasting stress treatments had detrimental effects during the initial stages due to clear symptoms of increased PCD and loss of embryogenic potential, as opposed to mild stresses. Despite both kinds of treatments slightly improving the production of ses, those obtained when mild stresses were applied were of better quality. Moreover, alterations in the metabolic profile of samples subjected to heat-shock treatments were detected. These metabolites include amino acids such as leucine, isoleucine, histidine and tyrosine and phenolic compounds, which could be involved in osmotic adjustment and antioxidative processes. These metabolic alterations reinforce the idea that long-lasting metabolic memory could endow future plants with an increased capacity to cope with adverse environmental situations.

## Author Contributions

PM and IM conceived and planned the experiments. AC-O, IM, and CP carried out the initiation experiments. AC-O, CP, NS, MG, and HF carried out the micromorphological study. AC-O, CP, EDM, MG, and HF carried out the ultrastructural analysis. AC-O, ED, LD’A, and PC carried out metabolite analyses. TG and MDU carried out the statistical analyses. AC-O wrote the manuscript and all authors provided critical feedback and helped to shape the research, analyses and manuscript.

## Conflict of Interest Statement

The authors declare that the research was conducted in the absence of any commercial or financial relationships that could be construed as a potential conflict of interest.
