# Supplementary material for: Effect of Thermal Stress on Tissue Ultrastructure and Metabolite Profiles During Initiation of Radiata Pine Somatic Embryogenesis
Source: Front Plant Sci. 2019 Jan 17;9:2004. doi: 10.3389/fpls.2018.02004 (PMC6344425; doi:10.3389/fpls.2018.02004)
Supplement: Supplementary file 1 [file Table_1.docx]

Supplementary Material

Effect of Thermal Stress on Tissue Ultrastructure and Metabolite Profiles During Initiation of Radiata Pine Somatic Embryogenesis

**Castander-Olarieta A.^1^, Montalbán I.A.^1ξ^, De Medeiros Oliveira, E.^2^, Dell’Aversana E.^3^, D’Amelia L.^3^, Carillo P.^3^, Steiner N.^2^, Fraga H.P.F.^4^, Guerra M.P.^2^, Goicoa T.^5^, Ugarte M. D.^5^, Pereira C.^6^, Moncaleán P.^1ξ^***

*** Correspondence:**Paloma Moncaleán
pmoncalean@neiker.eus

# Supplementary Tables

Table 1 Analysis of variance for the number of somatic embryos per gram of embryonal mass according to temperature (T).

| *Experiment 1* | | | | *Experiment 2* | | | |
| --- | --- | --- | --- | --- | --- | --- | --- |
| *Source* | *df* | *F value* | *p value* | *Source* | *df* | *F value* | *p value* |
| Temperature (T) | 2 | 3.1399 | 0.2081 | Temperature (T) | 3 | 0.30725 | 0.8198 |

Table 2 Analysis of variance for the amount of each metabolite according to temperature (T).

| Source: Temperature (T) | | | |
| --- | --- | --- | --- |
| *Variable* | *df* | *F value* | *p value* |
| Starch | 3 | 0.2664 | 0.8479 |
| Glucose | 3 | 0.4731 | 0.7095 |
| Fructose | 3 | 0.6372 | 0.6119 |
| Sucrose | 3 | 1.7803 | 0.1707 |
| Total protein | 3 | 0.0856 | 0.9660 |
| Alanine | 3 | 0.1068 | 0.9538 |
| Arginine | 3 | 1.0016 | 0.4405 |
| Asparagine | 3 | 0.5551 | 0.6591 |
| Aspartate | 3 | 1.3577 | 0.3232 |
| Ethanolamine | 3 | 1.0437 | 0.4243 |
| Phenylalanine | 3 | 1.0696 | 0.3758 |
| GABA | 3 | 1.1567 | 0.3415 |
| Glycine | 3 | 0.3786 | 0.7690 |
| Glutamate | 3 | 0.7953 | 0.5301 |
| Glutamine | 3 | 0.3765 | 0.7726 |
| Isoleucine | 3 | 5.8760 | 0.0026 |
| Histidine | 3 | 3.4462 | 0.0281 |
| Leucine | 3 | 1.0562 | 0.0001 |
| Lysine | 3 | 1.6620 | 0.1948 |
| Methionine | 3 | 0.6562 | 0.5850 |
| Ornithine | 3 | 0.0342 | 0.9914 |
| Proline | 3 | 1.0770 | 0.4120 |
| Serine | 3 | 0.3494 | 0.7909 |
| Tyrosine | 3 | 9.4533 | 0.0001 |
| Threonine | 3 | 0.7276 | 0.5637 |
| Tryptophane | 3 | 1.0492 | 0.3843 |
| Valine | 3 | 2.7466 | 0.1126 |

**
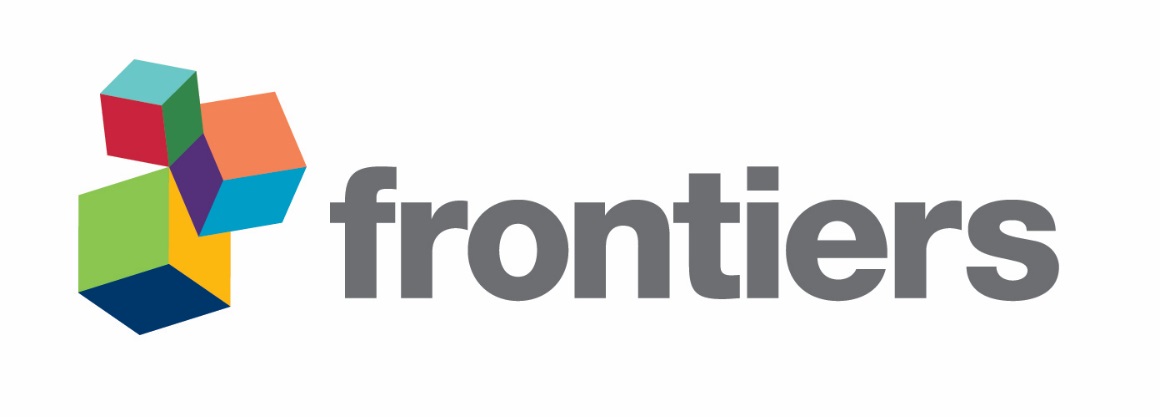
**
